# MicroRNA Library-Based Functional Screening Identified Androgen-Sensitive miR-216a as a Player in Bicalutamide Resistance in Prostate Cancer

**DOI:** 10.3390/jcm4101853

**Published:** 2015-10-20

**Authors:** Toshiaki Miyazaki, Kazuhiro Ikeda, Wataru Sato, Kuniko Horie-Inoue, Koji Okamoto, Satoshi Inoue

**Affiliations:** 1 Division of Gene Regulation and Signal Transduction, Research Center for Genomic Medicine, Saitama Medical University, 1397-1 Yamane, Hidaka-shi, Saitama 350-1241, Japan; E-Mails: tmiyaza@saitama-med.ac.jp (T.M.); ikeda@saitama-med.ac.jp (K.I.); wsatou@saitama-med.ac.jp (W.S.); khorie07@saitama-med.ac.jp (K.H.-I.); 2 Division of Cancer Differentiation, National Cancer Center Research Institute, 5-1-1 Tsukiji, Chuo-ku, Tokyo 104-0045, Japan; E-Mail: kojokamo@ncc.go.jp; 3 Department of Geriatric Medicine, Graduate School of Medicine, The University of Tokyo, 7-3-1 Hongo, Bunkyo-ku, Tokyo 113-8655, Japan; 4 Department of Anti-Aging Medicine, Graduate School of Medicine, The University of Tokyo, 7-3-1 Hongo, Bunkyo-ku, Tokyo 113-8655, Japan

**Keywords:** microRNA, hormone therapy resistance, androgen, prostate cancer

## Abstract

Prostate cancer is a major hormone-dependent tumor affecting men, and is often treated by hormone therapy at the primary stages. Despite its initial efficiency, the disease eventually acquires resistance, resulting in the recurrence of castration-resistant prostate cancer. Recent studies suggest that dysregulation of microRNA (miRNA) function is one of the mechanisms underlying hormone therapy resistance. Identification of critical miRNAs involved in endocrine resistance will therefore be important for developing therapeutic targets for prostate cancer. In the present study, we performed an miRNA library screening to identify anti-androgen bicalutamide resistance-related miRNAs in prostate cancer LNCaP cells. Cells were infected with a lentiviral miRNA library and subsequently maintained in media containing either bicalutamide or vehicle for a month. Microarray analysis determined the amounts of individual miRNA precursors and identified 2 retained miRNAs after one-month bicalutamide treatment. Of these, we further characterized miR-216a, because its function in prostate cancer remains unknown. miR-216a could be induced by dihydrotestosterone in LNCaP cells and ectopic expression of miR-216a inhibited bicalutamide-mediated growth suppression of LNCaP cells. Furthermore, a microarray dataset revealed that the expression levels of miR-216a were significantly higher in clinical prostate cancer than in benign samples. These results suggest that functional screening using an miRNA expression library could be useful for identifying novel miRNAs that contribute to bicalutamide resistance in prostate cancer.

## 1. Introduction

Prostate cancer is the second most common cancer among men worldwide and the incidence of prostate cancer has been increasing in Japan. Because the growth of prostate cancer is primarily regulated by androgen signaling, androgen deprivation therapy is often performed as prostate cancer treatment. The hormone therapy is initially effective for inhibiting the growth of prostate cancer by suppressing androgen receptor (AR) activity. Nevertheless, patients eventually acquire resistance to hormonal therapy during long-term treatment, and develop an advanced form of the disease, termed castration-resistant prostate cancer (CRPC) [1,2,3]. Patients with CRPC have a poor prognosis and account for the majority of deaths due to the disease.

Interestingly, recent studies have shown that AR signaling regulates prostate cancer growth even under the condition of androgen deprivation in CRPC. CRPC is commonly associated with increased AR signaling due to AR overexpression, AR mutation, transcription cofactor activation, AR phosphorylation, and other processes [4,5,6,7,8]. Indeed, overexpression of AR mRNA or protein is found in most cases of CRPC [6,7,8]. These findings suggest that AR plays a critical role in the development and progression of prostate cancer at both primary and CRPC stages [9,10,11,12,13]. However, the precise molecular mechanisms underlying the resistance to endocrine therapy and recurrence in CRPC remain to be studied, in terms of its key regulators and signaling events. As one of the new transcriptional regulators involved in cancer biology, the dysregulation of microRNAs (miRNAs) has been paid attention to various disease states including tumor progression, metastasis, and angiogenesis [14,15,16]. miRNAs function as transcriptional modulators by binding to complementary sequences in the 3′-untranslated region of their target mRNAs [17].

In the present study, we performed lentiviral miRNA library screening to identify novel miRNAs modulating the response to the anti-androgen bicalutamide in human prostate cancer LNCaP cells. By comparing the integrated miRNAs in the genomes of cells treated with bicalutamide and vehicle for one-month, two retained miRNAs were selected based on the fold change values of array signal intensities (by >5-fold). We focused on miR-216a, one of the retained miRNAs in the bicalutamide-treated cells, and examined its effect on the growth of LNCaP cells. Overexpression of miR-216a inhibited bicalutamide-mediated growth suppression of LNCaP cells. We found that miR-216a was overexpressed in long-term androgen-deprived bicalutamide-resistant LNCaP (LTAD-BicR) cells compared with parental LNCaP cells. Moreover, clinical prostate cancer samples showed higher levels of miR-216a expression than benign samples. These results show that miRNA library-based functional screening is useful for identification of novel miRNAs that are critical for bicalutamide responses in prostate cancer. These miRNAs could be applied for the development of alternative options for the diagnosis and treatment of prostate cancer.

## 2. Results

### 2.1. Screening for miRNAs Affecting Bicalutamide Responses in Prostate Cancer LNCaP Cells

**Figure 1:**
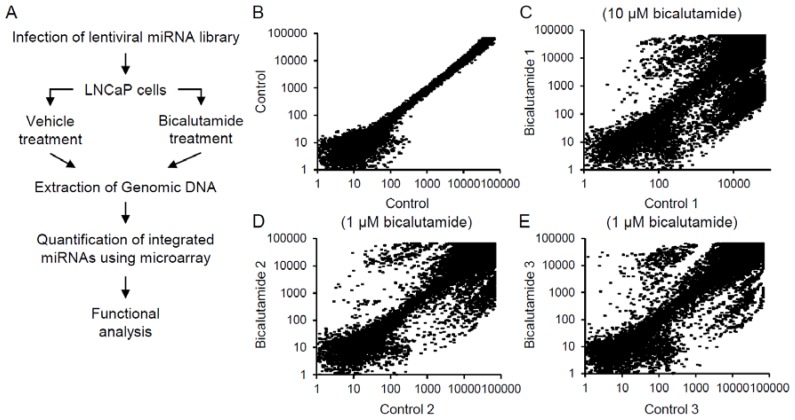
Screening of miRNAs associated with bicalutamide responses in LNCaP cells. (**A**) Schematic representation of screening procedure using a lentiviral miRNA library to identify mediators of the bicalutamide responses in human prostate cancer LNCaP cells. In brief, cells were infected with a lentiviral miRNA library and further cultured in regular media containing normal FBS with or without anti-androgen bicalutamide. Amounts of miRNAs integrated in the genomic DNAs of surviving cells were quantified by microarray; (**B**) Validation of miRNA screening reproducibility using two controls experiment is shown; (**C**–**E**) Scatter plots of array signal intensities for individual miRNAs for three groups of bicalutamide-treated and vehicle-treated samples ((**C**) 10 μM bicalutamide Sample 1 *versus* Control 1; (**D**) 1 μM bicalutamide Sample 2 *versus* Control 2; (**E**) 1 μM bicalutamide Sample 3 *versus* Control 3).

**Figure 2:**
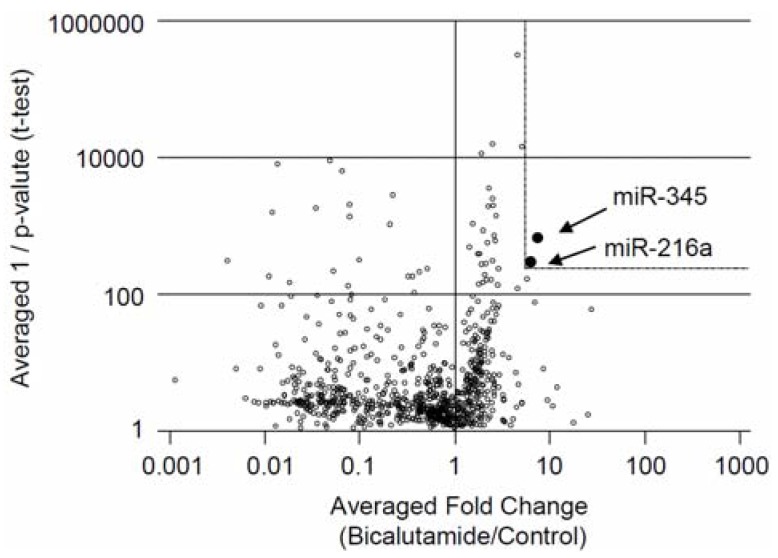
Volcano plot shows comparative analysis of miRNA microarray of the averaged three groups of the bicalutamide-treated cells and vehicle-treated cells. Volcano plot of microarray data generated by clustering based on probes that were retained (fold change >5; *p* < 0.01) in the averaged three groups of the bicalutamide-treated cells compared to vehicle-treated cells (Control). Closed circles represent selected miRNAs in this study.

To identify miRNAs involved in the bicalutamide responses in LNCaP cells, we performed functional screening with a lentiviral library comprising 445 miRNA precursors. LNCaP cells were infected with the library at different multiplicities of infection, and cell populations showing 30%–40% infection efficiency were selected, screened, and continuously cultured for one month in the presence of 1 or 10 μM bicalutamide or vehicle (Figure 1A). At the end of the cultivation period, genomic DNA was extracted from the surviving cells. The miRNAs that had integrated into the genome were amplified by PCR, using specific primers against the common sequences that flanked each miRNA, and then quantified by custom-made microarrays using the two-color of Cy-3 and Cy-5 fluorescent probe hybridization system. The array signal plots comparing the 2 control samples were linearly distributed along a diagonal line (Figure 1B), indicating that the biological duplicates exhibited high reproducibility. In contrast, plots comparing the bicalutamide-treated samples with the control samples were widely distributed. The upper left- or lower right-positioned plots separated by the diagonal line corresponding to the ectopic miRNAs that were retained or dropped out after one-month bicalutamide treatment, respectively (Figure 1C, 10 μM bicalutamide Sample 1 *versus* Control 1; Figure 1D, 1 μM bicalutamide Sample 2 *versus* Control 2; Figure 1E, 1 μM bicalutamide Sample 3 *versus* Control 3). Based on fold changes and *p* values (>5-fold at a threshold of *p* < 0.01), we identified two retained miRNAs that were upregulated in bicalutamide-treated cells (Table 1). The distribution of the retained miRNAs can be visualized by volcano plotting using averaged values for fold change and inverse *p* value as *x*- and *y*-axes, respectively (Figure 2).

**Table 1:** Retained miRNAs after bicalutamide treatment.

miRNA	Control ^a^	Bicalutamide ^b^	Bicalutamide/Control	*p* Value
miR-345	4737.4 ± 4127.3	32086.3 ± 4257.2	6.77	0.0013
miR-216a	7534.2 ± 7345.9	40038.2 ± 8824	5.31	0.0087

^a^ Averaged signal intensity of miRNA in the vehicle-treated control cells was quantified by microarray. The results are shown as mean ± S.D. ^b^ Averaged signal intensity of miRNA in the bicalutamide-treated cells was quantified by microarray. The results are shown as mean ± S.D.

### 2.2. miR-216a is Androgen-inducible and Overexpression of miR-216a Inhibits Bicalutamide-dependent Suppression of LNCaP Cell Growth

**Figure 3:**
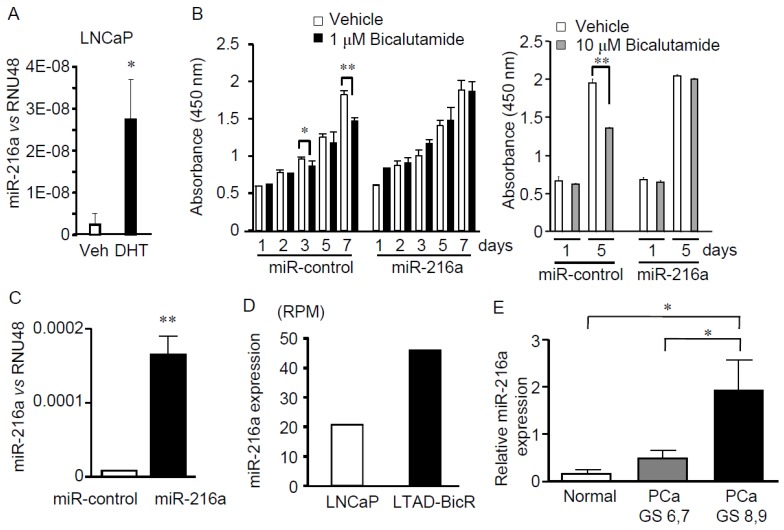
Overexpression of miR-216a inhibited bicalutamide-dependent suppression of LNCaP cell growth and upregulation of miR-216a in bicalutamide-resistant LNCaP cells and clinical prostate cancer samples. (**A**) Endogenous miR-216a expression is androgen-inducible in LNCaP cells. Cells maintained in hormone-deprived medium (phenol red-free medium with charcoal stripped FBS) were treated with 5α-dihydrotestosterone (DHT) (10 nM) or vehicle for 48 h and relative expression of mature miR-216a was determined by normalization to RNU48 expression evaluated by qPCR. Statistical analysis was performed using Student’s *t*-test. *, *p* < 0.05; (**B**) Overexpression of miR-216a inhibits bicalutamide-dependent suppression of LNCaP cell growth. Cells were infected with miR-216a precursor or miR-control, and then treated with 1 μM bicalutamide or vehicle in regular media with normal FBS. Cell proliferation was examined using WST-8 at indicated time points. The absorbance was read on a microplate reader at a wavelength of 450 nm. The results are shown as mean values ± S.D. (*n* = 4). Statistical analysis was performed using Student’s *t*-test. * *p* < 0.05; ** *p* < 0.01; (**C**) Relative expression of mature miR-216a on Day 7 after lentiviral transduction of miR-216a or miR-control was determined by normalization to RNU48 expression evaluated by qPCR. **, *p* < 0.01. Cells were cultured in regular media with normal FBS; (**D**) Small RNA sequencing using RNAs from LNCaP and LTAD-BicR cells shows that miR-216a expression was significantly upregulated in bicalutamide-resistant LTAD-BicR cells as compared to parental LNCaP cells. LNCaP and LTAD-BicR cells were maintained in regular media with normal FBS and phenol red-free media with charcoal-stripped FBS, respectively. The miRNA expression is quantified in terms of RPM (Reads Per Million) value, which is normalized against total reads in the sample; (**E**) Increased expression levels of miR-216a in advanced prostate cancer samples (Gleason score 8 and 9) compared with normal prostate samples or with lower-grade prostate cancer samples (Gleason score 6 and 7), based on an miRNA sequencing SDS144 dataset in The Cancer Genome Atlas. Relative miR-216a expression levels were calculated from original log2 values in the dataset. Normal prostate tissues, *n* = 4; prostate cancers with Gleason score 6 and 7, *n* = 23; and prostate cancers with Gleason score 8 and 9, *n* = 8. *, *p* < 0.05.

Alteration of miRNA expression may account for the change of bicalutamide resistance or sensitivity in LNCaP cells by modulating their target gene expression. Overexpressed miRNAs in cancers that promote oncogenesis are known as oncomiRs, whereas underexpressed miRNAs act as tumor suppressor miRs [18,19]. In this study, we focused on the retained miRNAs that could silence the expression of tumor suppressor genes. One of the retained miRNAs was miR-345, which has been reported to be associated with drug resistance and markers in cancers including breast and colorectal cancers [20,21]. miR-216a was further investigated because miR-216a is a candidate miRNA regulated by the androgen pathway [22]. We examined endogenous miR-216a expression in LNCaP cells and showed that the miRNA is upregulated by 5α-dihydrotestosterone (DHT) (10 nM) treatment (Figure 3A). To examine whether the miR-216a modulates bicalutamide resistance, LNCaP cells were infected with recombinant lentivirus that expresses the miR-216a precursor and subjected to the cell viability assay using 2-(2-methoxy-4-nitrophenyl)-3-(4-nitrophenyl)-5-(2,4-disulfophenyl)-2*H*-tetrazolium (WST-8). WST-8 assay revealed that ectopic miR-216a expression blunted the bicalutamide-mediated suppression of LNCaP cell growth, whereas bicalutamide treatment significantly repressed the growth of LNCaP cells infected with a control miRNA (miR-Control) (Figure 3B). Lentiviral transduction of miR-216a precursor elicited substantial ectopic expression of the mature miRNA in the cells at Day 7, as shown by qPCR (Figure 3C).

### 2.3. miR-216a is Upregulated in Bicalutamide-resistant LNCaP Cells and Clinical Prostate Cancer Samples

To determine the endogenous expression levels of the miR-216a, we generated bicalutamide-resistant cells, LTAD-BicR by long-term culture (>3 months) of LNCaP with bicalutamide and phenol red-free medium. Small RNA sequencing using RNAs prepared from LTAD-BicR and parental LNCaP cells showed that the expression levels of miR-216a were significantly upregulated in LTAD-BicR cells, as compared to parental cells (Figure 3D). We next examined the expression levels of miR-216a in clinical samples based on the miRNA sequencing dataset (#SDS144) retrieved from The Cancer Genome Atlas (TCGA) (Figure 3E). We found that the miR-216a expression levels were significantly higher in prostate cancers with Gleason score 8 and 9 compared to normal prostate tissues (*p* < 0.05), and also significantly higher in prostate cancers with Gleason score 8 and 9 compared to those with Gleason score 6 and 7 (*p* < 0.05). Taken together, studies on cultured cancer cells and clinical samples suggest that endogenous miR-216a expression associates with endocrine resistance and progression of prostate cancer.

## 3. Discussion

In the present study, we performed a functional screening using a lentiviral miRNA library to identify miRNAs associated with acquired resistance for endocrine therapy in prostate cancer. LNCaP cells infected with the miRNA library were treated with bicalutamide or vehicle for one month, and then the profiles of the genome-integrated miRNAs were compared. We identified two retained miRNAs in the bicalutamide-treated cells compared with the control cells. These miRNAs might be involved in the modulation of bicalutamide resistance in LNCaP cells. We focused on one of the upregulated miRNAs, miR-216a, and found that this miRNA is androgen-inducible. Then, we demonstrated that the overexpression of miR-216a significantly inhibited the bicalutamide-mediated growth suppression of LNCaP cells. We used 1 μM bicalutamide because it was shown that μM order of bicalutamide is a sufficient concentration for repressing the growth of LNCaP cells [23]. Our study showed that miR-216a expression increased in bicalutamide-resistant LNCaP cells, and an miRNA-seq dataset from TCGA also revealed the upregulation of miR-216a in clinical prostate cancer samples at advanced disease stages.

miR-216a is reported as an miRNA that is regulated by the androgen pathway at early stages of hepatocarcinogenesis. Consistently, we showed that miR-216a is an androgen-inducible miRNA as determined by our qPCR analysis. It is also notable that several androgen-dependent AR binding sites are located in the upstream genomic region of miR-216a as shown in the ChIP-on-chip data for prostate cancer [24]. Among them, the nearest AR binding site is located at ~6 kb upstream region of miR-216a and the sequence of the binding site contains at least five consensus androgen response elements analyzed by the JASPER open-access database of transcription factor binding profiles [25]. Thus, miR-216a could be an androgen target miRNA in prostate cancer cells. In addition, miR-216a targets tumor suppressor in lung cancer-1 (*TSLC1*), which modulates cell cycle progression, cell proliferation, and apoptosis [22]. Upregulation of miR-216a has been also reported in diabetic glomerular mesangial cells and this miRNA, together with miR-217, targets PTEN and activates AKT, leading to glomerular mesangial cell survival and hypertrophy [26]. In our screening of miR-216a target genes using several predicting programs (TargetScan, DIANA-microT, miRDB, and miRTarBase), we found that this miRNA could targets PTEN and TGFBR2. As loss of PTEN and TGFBR2 from prostate has been shown to result in castration-resistant cancer with metastases [27], we assume that miR-216a would play a critical role in the modulation of AR signaling and development of endocrine resistance. Future studies are required to clarify the precise role of miR-216a in prostate cancer.

We also identified miR-345 as another retained miRNA. miR-345 was found to be differentially expressed between breast cancer MCF-7 cells and the derivative cisplatin-resistant cells. In this report, miR-345 was demonstrated to target the multidrug resistance-associated protein 1 (MRP1) and suggested to be responsible for development of resistance to anticancer drugs [20]. In addition, miR-345 level in whole blood was a prognostic biomarker for overall survival and progression-free survival of patients with metastatic colorectal cancer treated with cetuximab and irinotecan [21]. These observations suggest that miR-345 may modulate drug resistance and could serve as a prognostic marker in cancers. Thus, miR-345 might be also involved in prostate cancer.

We recently reported of an miRNA library screen to identify miRNAs modulating tamoxifen responses in human breast cancer MCF-7 cells. By comparing miRNA expression in cells treated with 4-hydroxytamoxifen (OHT) to that in vehicle-treated cells, we successfully identified miR-574-3p as a modulating factor for tamoxifen response in breast cancer [28]. Indeed, miR-574-3p has been reported as a tumor suppressor in prostate, bladder, and gastric cancers [29,30,31]. The present study was also designed to identify miRNAs associated with anti-hormone resistance. We speculate that the results of these studies can provide new information regarding miRNAs that play critical roles in the development of hormone therapy resistance.

In summary, we showed that functional screening based on lentiviral miRNA library is useful for identifying miRNAs involved in bicalutamide resistance in prostate cancer cells. This approach could provide new targets for the diagnosis and treatment of advanced prostate cancer.

## 4. Materials and Methods

### 4.1. Screening of Lentiviral miRNA Library and Microarray Analysis

Experimental concepts of our screen method were based on previous literature [32]. Briefly, a human miRNA precursor lentivirus library that was consisted of a pool of 445 human miRNA precursor clones coexpressing GFP was purchased from System Biosciences (Mountain View, CA, USA). The library was infected to LNCaP cells with at different multiplicities of infection together with 5 mg/mL polybrene. Transduction efficiency was evaluated by GFP expression 48 h after infection using FACS Calibur (Becton Dickinson, CA, USA).

To avoid the possibility of multiple infections, we selected cell populations with 30 to 40% infection efficiency. Cells were continuously cultured in RPMI medium containing 1 or 10 μM bicalutamide or vehicle for 4 weeks. During the culture period, medium was replaced every 2 to 3 days. Then, miRNA precursors integrated into the surviving cells were amplified by PCR using specific primers against the sequences in the lentivirus vector (forward primer: 5′-GCCTGGAGACGCCATCCACGCTG-3′; reverse primer: 5′-GATGTGCGCTCTGCCCACTGAC-3′), in order to amplify miRNA precursor sequences. PCR products from bicalutamide-treated and vehicle-treated LNCaP cells were labeled with Cy-3 or Cy-5, respectively, using the Genome DNA Enzymatic Labeling Kit (Agilent Technologies, Santa Clara, CA, USA) and then subjected to microarray hybridization (Oligo cDGH/ChIP-on-ChIP Hybridization Kit, Agilent Technologies). Agilent Feature Extractor software was used to scan microarray images and to normalize signal intensities. A volcano plot was generated by clustering based on probes. Signals (fold change >5; *p* < 0.01) in the averaged 3 groups of the bicalutamide-treated LNCaP cells compared to vehicle-treated cells were selected as candidate miRNAs potentially involved in the bicalutamide resistance.

### 4.2. Cell Culture and Transduction of miRNA Precursors by Lentiviral Vector

LNCaP prostate cancer cells were purchased from the American Type Culture Collection (Manassas, VA, USA). LNCaP cells were cultured in RPMI supplemented with 10% fetal bovine serum, penicillin (50 U/mL), and streptomycin (50 μg/mL) at 37 °C in a humidified atmosphere containing 5% CO_2_. Long-term androgen-deprived bicalutamide-treated cells (LTAD-BicR cells) were established from LNCaP cells by long-term (>3 months) treatment with 1 μM bicalutamide in phenol-red free RPMI supplemented with 10% charcoal-dextran-treated fetal bovine serum, penicillin (50 U/mL), and streptomycin (50 μg/mL) at 37 °C in a humidified atmosphere containing 5% CO_2_. Transduction of miR-216a precursor or control miRNA (Pre-miR™ miRNA Precursor, Life Technologies, CA, USA) into LNCaP cells were carried out by generating virus-containing supernatants as previously reported [32]. Briefly, lentivirus plasmids were co-transfected with pLP1, pLP2, and pLP/VSVG (Invitrogen) into 293FT cells (Invitrogen), and virus containing supernatants were prepared according to manufacturer’s instructions. For infection, cells were incubated with virus-containing supernatants in the presence of 6 mg/mL polybrene.

### 4.3. RNA Extraction and High-throughput Sequencing

Total RNAs were isolated from LNCaP and LTAD-BicR cells using the ISOGEN reagent (Nippon Gene, Toyama, Japan) in accordance with the manufacturer’s instruction. Small RNA cDNA library was generated from the total RNAs and high-throughput sequencing was performed using an Illumina GAIIx sequencer (Illumina, San Diego, CA, USA) [33]. Mapping of small RNA reads were performed on human genomes (NCBI35 assembly). In quantitative PCR (qPCR) experiments, miRNA levels in LNCaP cells were determined by StepOne Real-time PCR System (Applied Biosystems) using TaqMan microRNA assays (Applied Biosystems, CA, USA). Results from three independent experiments were normalized to the expression of endogenous RNU48. 5α-Dihydrotestosterone (DHT) (Nacalai Tesque, Kyoto, Japan) treatment was performed at 10 nM concentration for 48 h.

### 4.4. Cell Growth Assay

The effects of bicalutamide or miRNAs on cell viability were determined by the WST-8 assay using the Cell Count Reagent SF (NACALAI TESQUE, Kyoto, Japan). LNCaP cells were lentivirally transduced with miR-216a or miR-control and were seeded into 96-well plates at a density of 5000 or 3000 cells per well, and 10 μL of WST-8 solution was added to each well at the indicated time points (24, 48, 72, 120 or 168 h) after transfection. Cells were further incubated for 3 h at 37 °C in a 5% CO_2_ incubator. The absorbance was measured at 450 nm with Multiscan FC Microplate Photometer (Thermo Fisher Scientific, MA, USA). The results were shown as mean ± S.D. (*n* = 4). Statistical analysis was carried out using Student’s *t*-test. *, *p* < 0.05; **, *p* < 0.01.
